# Indiana State Dental Society

**Published:** 1872-10

**Authors:** B. P. McDonald


### PROCEEDINGS OF SOCIETIES.

### Indiana State Dental Society.

The fourteenth annual meeting of this society was held at Indianapolis, June 25th, 1872.

The President, Dr. P. G. C. Hunt, in the chair, with a fair representation of the members of the association in attendance.

The meeting was called to order at 2 o'clock, P. M. When the minutes of the last annual meeting were read and approved.

Several visiting dentists were present and were made honorary members of the Association. Drs. H. J. McKellops, of St. Louis, Mo.; G. V. Black, of Jacksonville, Ill.; Prof. J. Taft, of Cincinnati, Ohio, and many others.

The chair appointed as the Board of Censors, Prof. A. M. Moore, Drs. J. W. Ellis and H. T. Manlove.

The follwing persons having passed a satisfactory examination before the board, were by ballot elected members of the Association: Drs. L. J. Mason, of Fort Wayne; S. E. Harryman, of Lawrenceburg, and F. M. Hamsher, of La Grange.

The Treasurer being absent, Dr. John F. Johnson, of Indianapolis, was appointed Treasurer *pro tem.*

The President delivered the annual address which elicted the closest attention from the association.

By motion of Prof. Richardson the physicians of Indianapolis were cordially invited to attend the sessions of the association.

The subjects before the association being in order, on motion of Dr. Johnson the eight subject was selected for discussion; viz: "Broken Points for Pluggers," which elicited an animated discussion from Drs. Hunt, Black, Morrison, Moore and Richardson—all more or less favoring this new era in the improvements of filling instruments.

Dr. Black stated that he had used broken pluggers to excellent advantage by grinding off the broken or crystalized surface, and using the instrument as it came from the corrundum wheel: light numbers of foil weld more perfectly in the use of this instrument.

By consent the eighth subject was closed, and the ninth opened for discussion, viz: "Base for artificial teeth, best substitute for rubber." Considerable attention was given this subject, yet arriving at no unanimous conclusion concerning a substitute for rubber. "Celluloid Base" was mentioned as perhaps a suitable substance to take the place of rubber for cheap dentures, yet the introduction of this base has been of so recent a date that a proper test has not been arrived at in relation to premanency.

The association expressed the opinion however, that the day was not far distant when a substance would take the place of rubber, and other cheap materials, far better and purer in every particular.

The hour for adjournment being nearly at hand, President Hunt appointed the following gentlemen to conduct the clinic on the following morning; Prof. J. Richardson; Drs. H. J. McKellops and J. V. Black. Adjourned.

MORNING SESSION, SECOND DAY.

The President called the association to order, the minutes of last meeting read and concurred in.

Prof. Richardson and Dr. McKellops failed to procure a case suitable for clinic.

Dr. Black operated upon a left inferior molar, cavity posterior proximal surface. Whereupon he illustrated the efficiency of his dental engine—being of similar construction to the justly celebrated "Morrison Engine." Dr. Black is deserving of great praise for the introduction of this important auxiliary to facilitate tedious operations; and very clearly demonstrated to the association the ease with which he prepared the cavity for filling, and the exquisite finish it produced upon the filling after it was completed. This instrument with the rubber dam are now indispensable to the profession, and so acknowledged by the association.

After the clinic was concluded the first subject for discussion was taken up, viz: The essential principles in filling teeth."

President Hunt in his pleasant and forcible manner gave the association the benefit of some beautiful ideas on this subject, and no doubt inspired the younger members of the association with renewed vigor for the grand opportunities that are now being offered in this direction.

Dr. Hunt also exhibited several cases of models of successful ones of filling, or restoring the contour of the teeth with gold after the crowns had been lost by decay or attrition.

Prof. Moore also exhibited a mould of a very extensive case of building on, or restoring the teeth (*both above and below, sixteen in number*) to their normal formande size. This was a very extensive case, and evidently was an exceeedingly wearisome one.

Two much praise can not be said of these two cases (Dr. Hunt's and Prof. Moore's) as they involved most of the theories and practices that are now being urged by the best and ablest men in the profession.

Prof. J. Taft and Richardson, Drs. W. H. Goddard, of Louisville, Ky., and H. A. Smith, of Cincinnati, Ohio, expressed great interest in this subject, and valuable ideas were exchanged for the remainder of the morning, which concluded the first subject. Adjourned.

The association met persuant to adjournment at 2 o'clock P. M.

President Hunt in the chair. Reading the minutes of last meeting were dispensed with. The Secretary being absent Dr. E. M. Morrison was appointed Secretary, pro tem.

Dr. Keightly moved that Dr. W. F. Morrill's paper be read, Concurred in.

On motion, Dr. E. M. Morrison, of Noblesville, was requested to read his paper.

Prof. Moore moved that the papers of Dr. W. F. Morrill on "Fine Art in Dentistry," and Dr. E. M. Morrison on "The lying down posture in the administration of anæsthetics," and the President's address be referred to the Publishing Committee

The State Superintendant of the Hospital for the Insane, extended an invitation to the association, through Dr. John F. Johnson, to visit the hospital. Whereupon the thanks of the association were tendered the Superintendant for his kind consideration, and the compliment acknowledged.

By motion of Dr. E, M. Morrison, the association proceeded to the election of officers for the ensuing year; with the following result: President—Pɪ of. Joseph Richardson, of Terre Haute; First Vice President.—Drs. E. M. Morrison, of Noblesville; Second Vice President—H. T. Manlove, Logansport; Treasurer—John F. Johnson, Indianapolis; Secretary—B. P· McDonald, Goshen.

The President Elect was conducted to the chair, by Prof. A. M. Moore and Dr. S. B. Browne, where in a brief speech he thanked the members for the honor conferred upon him.

Prof. J. Taft, was requested to favor the association with an explanation of the law regulating the practice of dentistry. in the State of Ohio. To which he acceeded, explaining its good effect, manifested in various ways.

Also Dr. H. A. Smith, of Cincinnati, was called upon to report. He also gave a very satisfactory account of the good work, legislation will do for the country—protecting the people from imposing charlatans, also educating the deserving though poor dentist to a better standard of operations. Dr. Smith was very earnest in his statements, and proved himself familiar with the great good this legislation has brought about, over the entire State of Ohio.

After a discussion for sometime upon this subject, and being well and earnestly advocated, a motion was made that a committee of five be appointed to memorialize the legislature of Indiana, for the purpose of procuring a dental law. Concurred in.

The association appointed as the Committee on Legislation Drs. P. G. C. Hunt, Chairman; John F. Johnson, Indinapolis; Prof. A. M. Moore, Lafayette; S. B. Brown, Fort Wayne, Prof. J. Richardson, of Terre Haute.

Dr. E. M. Morrison moved that the Committee on Legislation be empowered to draw on the Treasurer of the association for all necessary funds. Accepted.

The President appointed as Auditing Committee, Drs. E. M. Morrison and Merit Wells.

By special action the time of the next annual meeting of the association was changed from the last Teusday in June to the first Tuesday in June of next year.

In order that the Indiana Dental Society may meet at the same time with the Kentucky Dental Society (which convenes at Louisville,) and in order to make the meetings more interesting, each alternate day one society would meet with the other; therefore Dr. Keightley moved that the place of meeting for next June should be at New Albany. Adopted. Adjourned.

MORNING SESSION, THIRD DAY.

Association met at 9½ o'clock. The President being absent, Dr. E. M. Morrisson was appointed pro tem. The minutes of last meeting were read, corrected and approved.

The second subject was taken up for discussion, viz: "Dr. Arthur's system of prevention and treatment of decay of the teeth."

Prof. Taft thought the book of Dr. Arthur was in many respects a good one, and evidently written with care and great earnestness. The author had no doubt perfected himself in this specialty so thoroughly that he has accomplished what he desired, viz: the removal of decay, and the saving of teeth. Yet he thought it was a book that would do a great amount of mischief if it was accessible to the public; however, every dentist should read it carefully, and would no doubt be profited thereby.

Dr. McKellops looked with favor upon Dr. Arthur's system, in many particulars, yet could not subscribe in full to his tenets. He advised a thorough reading of the work, and perhaps a second or third reading.

Dr. Hunt was of the opinion that this system was very much overrated. He had seen some of Dr. Arthur's work in treatment of decay, and to him it presented a very sad spectacle. Dr. Arthur had separated the teeth (those that came under his observation), so they presented very much the appearance of teeth in a saw.

Dr. Hunt said his idea of what was done in this case would soon be in as bad or worse condition than when Dr. Arthur first operated upon them. And if Dr. Arthur would turn his attention to the filling of teeth as attentively as he has of late to his system of chiseling, he would accomplish a much greater good, as the filling of teeth has taken a new departure, and accomplished magnificent success everywh ere from the hands of skilled operators.

Others of the association took part in the discussion, and great interest was manifested. Many advocated the plugger over Dr. Arthur's system, as the great improvements of to-day placed it in the power of nearly *every dentist* to accomplish a success, where heretofore a failure was inevitable; but with rubber dam nothing but success is the common result.

By consent the subject was set aside, and the fifth chosen: "Dental Irregularities, best age for treatment." By invitation Drs. G. F. Canine an.l W. H. Goddard, of Louisville, Ky., illustrated to the association, by the use of models, how a very irregular set of teeth were by gentle treatment made to conform to the perfect law of nature. This case was a very interesting one, and attracted considerable attention.

Dr. Canine also, in connection with his models, presented for examination the different plates worn by the patient during the process of regulating.

This case clearly illustrated how an almost deformed mouth can be made beautiful, as far as the teeth are concerned. Every one present praised this case of Dr. Canine's, and expressed a desire that the regulating of teeth might become more general.

Dr. G. W. Keely, of Oxford, exhibited some successful models of cases he had regulated; also several others were brought forward for examination, by different members of the association.

The President appointed, as the Committee on Executive Business, Drs. Morrill, Driscoll and Johnston. Adjourned.

AFTERNOON SESSION.

The association met at 2½ o'clock, with President Richardson in the chair. The Secretary being absent, Dr. John F. Johnston was appointed Secretary pro tem.

The time for adjournment having nearly arrived, on motion the further discussion of the regular subjects was waived, and miscellaneous business taken up.

Dr. Johnston presented to the association an interesting report of cases of ossified pulps, by Prof. S. P. Cutler, of Holly

Springs, Miss. The report was read and referred to the Committee on Publication.

The thanks of the association were tendered Prof. Cutler, and the Secretary ordered to make known to him the same.

Dr. W. E. Driscoll read an essay, entitled "Thoughts on Success in Dental Practice." On motion the paper was received, and referred to the Committee on Publication.

Dr. Goddard, of Louisville, returned thanks in neat and appropriate terms, for the courtesies and kindness exhibited toward the visiting members of the profession by the association.

President Richardson responded in behalf of the association, alluding briefly to the warm interest exhibited on their part, and to the valuable information received, regarding it as more than an offset.

On motion a vote of thanks was tendered the Faculty of the Indiana Medical College for the use of their lecture room. Also, on motion of Dr. Hunt, to Strong and Smith for courtesies extended.

At the suggestion of Dr. E. M. Morrison, of Noblesville, Ind., a vote of thanks was tendered to the profession in this city who attended the meetings, and for many courtesies extended visiting members.

On motion of Prof. Richardson, a resolution of thanks was tendered to our worthy ex-President for the elegant and hospitable entertainment given to them at his residence.

On motion of Dr. Johnston a vote of hearty thanks was tendered Dr. O. Everts and his staff of assistants; Drs. Hoster and Ellston for their kind reception and attention in exhibiting the interior work and order of our noble charity, the hospital for the insane.

Thanks were voted the visiting members for their attendance, valuable suggestions, and kind counsel.

Several bills were presented and ordered to be paid.

By motion the association adjourned, to meet in New Albany on the first Tuesday in June, 1873.

B. P. McDonald,

*Secretary.*

